# Passage of Time Judgments Are Not Duration Judgments: Evidence from a Study Using Experience Sampling Methodology

**DOI:** 10.3389/fpsyg.2016.00176

**Published:** 2016-02-19

**Authors:** Sylvie Droit-Volet, John Wearden

**Affiliations:** ^1^Laboratoire de Psychologie Sociale et Cognitive, UMR 6024, CNRS, Université of Clermont AuvergneClermont-Ferrand, France; ^2^University of KeeleKeele, UK

**Keywords:** time, time perception, passage of time, Experience Sampling Methodology, elderly

## Abstract

This study examined relations between passage of time judgments and duration judgments (DJs) in everyday life, in young and elderly people, with an Experience Sampling Method. The DJs were assessed by verbal estimation and interval production measures. The results showed no difference between young and elderly people in judgments of rate of passage of time, a result contrary to the conventional idea that time passes more quickly as we get older. There were also no significant relation between the judgment of passage of time and the judgments of durations. In addition, the significant predictors of individual differences in the judgment of passage of time (emotion states and focus of attention on the current activity) were not predictors of judgment of durations. In sum, passages of time judgments are not related to DJs.

## Introduction

The main aim of the work reported in this article is to explore relations between passage of time judgments (PoTJs) and duration judgments (DJs), with the latter being assessed by verbal estimation and interval production measures. In addition, the present study provides a partial replication of Droit-Volet and Wearden’s (2015) recent work on a comparison of PoTJs in a student-age group and a group of elderly people, around 50 years older.

A PoTJ is a judgment about how fast time seems to pass in some situation. It is generally measured with a question such as “How fast does time pass for you?” or “How did time pass relative to clock time?”, with responses usually being given on a Likert-type scale with 7 points from “very slowly” to “very fast” (e.g., Blewett, 1992; Wittmann and Lehnhoff, 2005; Friedman and Janssen, 2010). Studies using this type of question have suggested that subjective experience of passage of time changes as a function of health status of individuals or contexts (for a review see Droit-Volet, 2013). For example, depressed people experience a slowing down of time passage, such that “time seems to drag. A day feels like a year” (Ratcliffe, 2012).

Some authors assume that this experience of time passage reflects fundamental changes in basic mechanisms underlying the representation of duration, for example, in the rate of the pacemaker of some sort of internal clock (Rammsayer, 1990). According to the popular Scalar Expectancy Theory, the judgment of durations depends on the number of pulses emitted by a pacemaker and counted in an accumulator (Gibbon, 1977; Gibbon et al., 1984). Consequently, if there is a slowdown of the internal clock rate in patients suffering from depression, they would introspectively feel this change, and would express it in terms of slowing down of the passage of time. However, as discussed later, in human beings, the consciousness of time passing faster or slower than usual cannot be simply dependent on number of pulses counted during an event. In line with this idea, a significant number of studies suggested that patients with depression do not show deficits in their abilities to judge stimulus durations, even though they experience time passing slowly (for a recent meta-analysis see Thönes and Oberfeld, 2015).

The principal question addressed here concerns this relation between PoTJs and judgments of duration. One way of addressing this issue might be to take concurrent measurements of both the judgment of durations and the judgment of passage of time and examine how the two measures covaried. A small number of studies have examined the relation between the retrospective judgment of durations (when the people were not alerted in advance that a question about time would be asked) and the judgment of the rate of passage of time. When participants were asked about PoTJs and DJs after an action film or a relaxation video, Wearden (2005) did not find any relationship between these two forms of judgment. In fact, people judged that time was passing more quickly for the action film than for the relaxation film, while the action film was retrospectively judged as slightly longer. Likewise, Wearden (2008) obtained PoTJs and retrospective time judgments of three durations of a film under two processing conditions. In one of these, the instruction was simply to watch the film and in the other people were required to count each time one of the characters had spoken after another character. The different conditions changed PoTJs, but DJs were unaffected.

However, the lack of relationship between the DJs and PoTJs in these studies might be related to the fact that, in the retrospective timing tasks used, the participant’s attention is not focused on the processing of time. In addition, the PoTJ and the DJ are measured from a single time interval, or a few time intervals, ranging from a few 1 seconds to minutes. Also, the PoTJ of an event created in laboratory may differ from PoTJs in everyday life which may involve longer periods of time. Lamotte et al. (2014) showed that individuals are not aware of most of the factors that influence their perception of durations in the range from milliseconds to seconds. They are only aware of fluctuations of passage of time in two contexts those when, introspectively, they feel themselves happy or sad and engaged in a daily activity. In a recent study assessing PoTJs in everyday life with the technique of *Experience Sampling Methodology* (ESM), Droit-Volet and Wearden (2015) found that PoTJs in everyday life changed with the individuals’ moods and their degree of immersion in the activity they were currently engaged in. Consequently, we decided to use the ESM method to examine the relation, if any, between prospective judgment of durations and the lived experience of passage of time, when the individuals are aware of their states of happiness and the intensity of their engagement in activity.

The ESM method (see Conner et al., 2003) is an ecological approach that is mainly used in clinical psychology but has been used in the domain of time psychology in a few studies (Conti, 2001; Larson and von Eye, 2006; Droit-Volet and Wearden, 2015, and recently Droit-Volet, in press a). Conti (2001) examined the link between PoTJs and intrinsic or extrinsic work motivation, and Larson and von Eye (2006) the link between this time judgment and the degree of engagement in activities. Droit-Volet and Wearden (2015) studied differences in PoTJs in young and elderly people. In all these ESM studies, the participants received “alerts,” at quasi-random times, several times per day (8 or 10). When an alert is received, the participant’s task is to respond to a short questionnaire, with the aim being to collect immediate impressions, rather than those based on reflection. For example, Droit-Volet and Wearden (2015) assessed PoTJs in young and elderly people at the time of the alert, as well as the rating of their emotional states (happiness, sadness, arousal, relaxation), and their level of occupation (the difficulty of the activity performed at the time of the alert – Activity difficulty – and the focus of attention on that activity – Attention capture –).

In the present ESM study, we thus assessed PoTJs, as well as people’s emotional states and the intensity of their activity, but we also measured DJs using a verbal estimation task, and an interval production task. This enabled us not only to relate PoTJs at the time of the alerts to DJs, but also enabled us to explore potential predictors of both PoTJs and DJs. In addition, following Droit-Volet and Wearden (2015) we used two participant groups, one of young people and the other of elderly persons.

## Materials and Methods

### Participants

The final sample was composed of 27 participants (18 women and 9 men): 14 students at University Clermont Auvergne (mean age = 20.50, *SD* = 1.99, age range from 18.88 to 22.42) and 13 elderly people (mean age = 69.46, *SD* = 3.62, age range from 68.01 to 71.42). Three additional individuals (one young and two elderly adults) participated in the study but stopped the experiment, so their data were not included in the final sample. All participants signed a consent form approved by the Sud-Est VI Statutory Ethics committee, and received 40 euros for their participation. The Mini Mental State Examination (MMSE) was administered to elderly people to reject those suffering from dementia (mean score = 29.54, *SD* = 0.66). The IQ scores on the Weschsler Adult Intelligence Scale (WAIS-III, Grégoire and Wierzbicki, 2009) were also measured but they did not differ significantly between the young (*M* = 98.28, *SD* = 10.70) and the older participants (*M* = 105.69, *SD* = 11.70), *t*(25) = -1.72, *p* = 0.10. Their depression scores on the Beck Depression Inventory (BDI) test (Beck et al., 1961) were also similar [3.64 vs. 3.23, *t*(25) = 0.29, *p* = 0.78].

### Apparatus

Motorola G Android Jelly Bean smartphones were used for this experiment with data collection programs specifically written by the CATech (http://lapsco.univ-bpclermont.fr/catech) of the Laboratory of Social and Cognitive Psychology of the University Clermont Auvergne. The programs delivered and recorded all experimental events (alerts, questions and temporal tasks). The participants responded by pressing on the touch screen of the smartphone. The stimulus used in the verbal estimation and the temporal production task was a sound (La, 440 hz).

### Procedure

Participants initially received an explanation of the procedure of the experiment and the functioning of the smartphone (with one demonstration), then filled in the different scales (MMSE; WAIS-III, BDI). They then kept their smartphone for five consecutive weekdays, from Monday to Friday. They received eight alerts per day, from 8.00 a.m. to 8.00 p.m., with one alert randomly delivered within each 90-min period, with at least 15 min between any two alerts. This made a total of 40 alerts per participants.

After each alert, the participant performed the verbal estimation task and the production task followed by their PoTJ. For the verbal estimation task, the participant was required to judge (using a scale ranging between 100 and 2000 ms) three different durations, each randomly chosen between (1) 350 and 650 ms, (2) 850 and 1150 ms, and (3) 1350 and 1650 ms, respectively. The presentation order of these three durations was random. For the production task, the participants were initially presented with a duration value: 500, 1000, or 1500 ms. A blue circle then appeared and the participants were required to press on the circle to trigger a sound, then release the pressure, thus stopping the sound, when they judged that the sound duration equalled the value indicated. The duration presentation order was also random. For all temporal tasks, the participant initiated a trial by touching the screen after the word “ready,’ and this was followed after 500 ms by the events of the trial. Furthermore, the participant did not receive feedback regarding their performance on the verbal estimations and time productions.

After the temporal tasks had been performed, the question “At the moment, the moment of the alert, how does time pass for you compared to the time of the clock” was given. The participant responded on a 7-point scale: “(1) much slower – (2) moderately slower – (3) a little slower – (4) at the same rate than the clock – (5) a little faster – (6) moderately faster – (7) much faster.” Following this PoTJ question, they responded to affective and activity questions. There were four affective questions: “At the moment of the alert, do you feel (1) happy” (Happiness), (2) “sad” (Sadness), “excited/stimulated” (Arousal) and “relaxed/calm” (Relaxation). The activity questions concerned the difficulty of the activity performed at the moment of the alert (Activity difficulty) and whether it captured the participants’ attention (Attention capture). For these different questions, participants responded on 7-point scale from “not at all” to a “lot.”

## Results

For the ESM phase of our study, the overall average percentage of missed alerts for the 40 alerts (8 alerts × 5 days) was 5 (*M* = 5, *SD* = 5.50) (min = 0, max = 20%) with no difference between the young and elderly participants on average [4.82 vs. 5.19, *t*(25) = 0.17, *p* = 0.87]. Consequently, nobody was excluded on the basis of their ESM scores. The responses for the different variables at the times of the alerts were analyzed by multi-level modeling, using SPSS. The multi-level modeling procedure is a variant of regression, which generates an estimate (coefficient) indicating whether the outcome variable and its predictor are positively related (with a positive coefficient) or negatively related (with a negative coefficient). The coefficient essentially represents how much the outcome variable changes for an unit change in the predictor. The significance of the relation between the variables is indicated by the significance of the predictor, assessed by a *t*-value. However, the values of the coefficients are not meaningful when the predictor variables are arbitrarily encoded, as for our between-group analysis. It is also important to recall that multi-level modeling is a type of repeated-measures design, and so is generally considered more powerful than a simple between-groups design (Bolger et al., 2012), especially when many observations per participant are collected for each variable measured (e.g., usually 40 in our study). Calculation of statistical power for multi-level designs is not, however, straightforward and disagreement exists as to the best method (Bolger et al., 2012; see also Nezlek, 2012).

**FIGURE 1:**
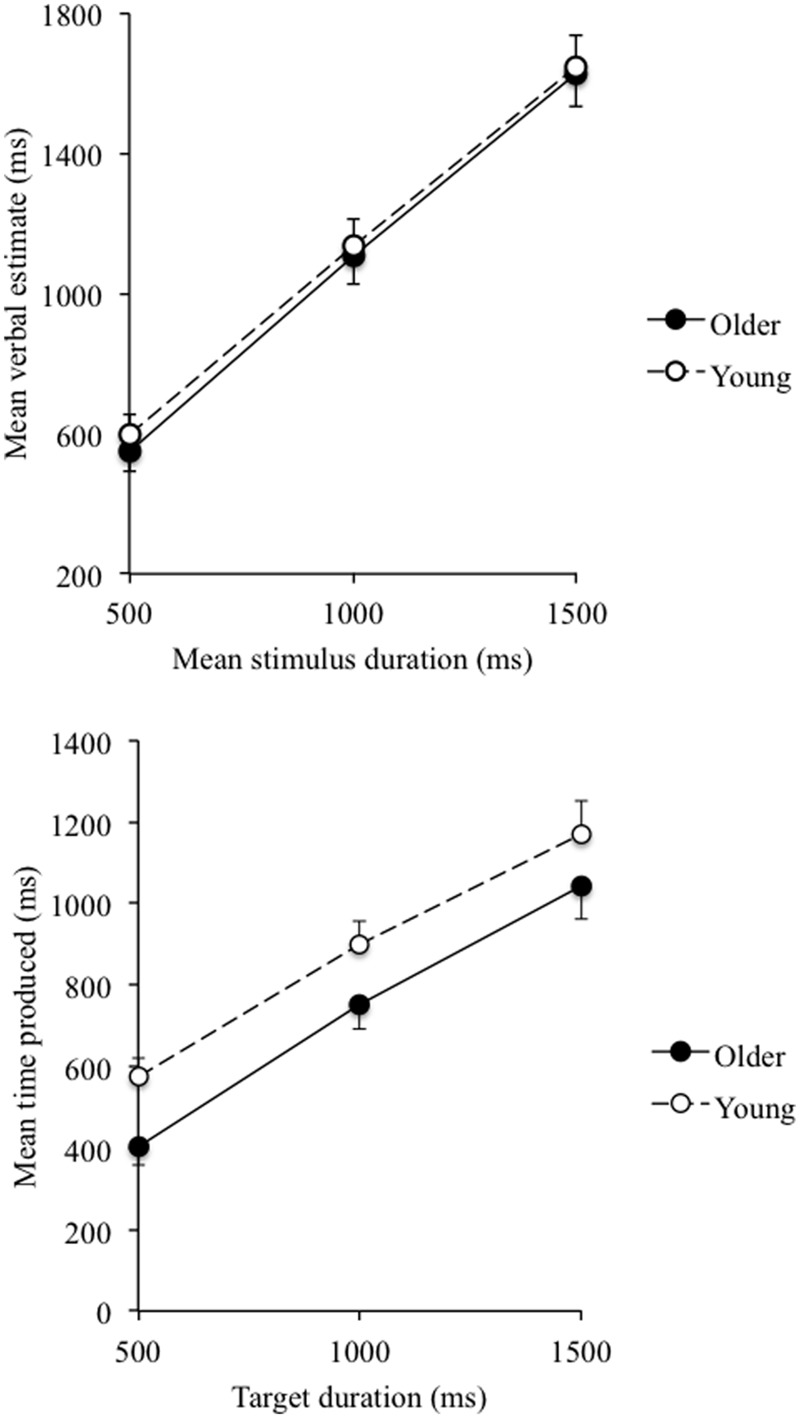
**Mean verbal estimates **(upper)** and mean time produced **(bottom)** plotted against stimulus duration in young and elderly people**.

**Table 1** shows means and standard errors of scores on the different variables tested in our study for the young and older groups. The *t-*values indicated the between-group differences on these variables. There were no significant between-group differences for the verbal estimates (all *p* > 0.05). A significant between-group difference was only observed for the temporal production of the shortest duration of 500-ms (*p* = 0.02). As shown in **Figure 1**, durations produced were shorter for the elderly group than for the young people when they had to produce the target duration of 500 ms. The between-group difference did not reach significance for two longer target durations. Additional analyses were performed with the day and the time of alert introduced into the model. These analyses showed no variation either in the verbal estimates or the durations produced with the time of the alert [verbal estimates: 500-ms, *t*(73.89) = 0.41; 1000-ms, *t*(65.45) = 0.53; 1500-ms, *t*(68.04) = 1.04; Production: 500-ms, *t*(34.24) = 1.16; 1000-ms, *t*(36.71) = 0.38; 1500-ms, *t*(36.41) = 0.37, all *p* > 0.05]. There was nevertheless a significant link between day and the verbal estimates for all stimulus durations [500-ms, *t*(130.47) = -4.10; 1000-ms, *t*(102.10) = -4.96; 1500-ms, *t*(78.49) = -3.82, all *p* = 0.0001], and the production of the different target durations [500-ms, *t*(35.45) = 3.16; 1000-ms, *t*(37.91) = 4.80; 1500-ms, *t*(35.60) = 4.96, all *p* < 0.001]. When we used a composite measure for the verbal estimates (mean of estimates for the three stimulus durations), the difference in verbal estimates reached significance between the first (1298.51) and the three last days (1078.77, 1025.11, and 996.25, respectively), and the second (1178.40) and two last days (Bonferroni, *p* < 0.05), with no difference between the two first days (*p* > 0.05). That is, the length of verbal estimates decreased over days. Conversely, for the temporal productions, the values of reproduced durations increased from the first to the third days (Bonferroni, *p* < 0.05) after which the time produced no longer changed (*p* > 0.05).

**Table 1:** Means and standard errors of measures of duration judgments from the verbal estimation and production tasks, scores on the passage of time judgments, and the other measures assessed by the ESM procedure.

Variable	Mean	Standard Error	*t*-value	*p*-value
**500-ms verbal estimation**				
Young	589.58	55.76	0.51	0.62
Older	548.88	57.83		
**1000-ms verbal estimation**				
Young	1135.64	77.29	0.25	0.80
Older	1107.46	80.17		
**1500-ms verbal estimation**				
Young	1646.10	89.90	0.14	0.89
Older	1627.75	93.25		
**500-ms production**				
Young	573.60	45.68	2.58	0.02
Older	403.94	47.39		
**1000-ms production**				
Young	898.63	58.77	1.72	0.10
Older	753.11	60.98		
**1500-ms production**				
Young	1171.38	79.34	1.17	0.25
Older	1044.27	82.33		
**Passage of time judgment**				
Young	4.75	0.20	0.90	0.38
Older	4.49	0.20		
**Happiness**				
Young	4.78	0.17	0.95	0.35
Older	5.01	0.18		
**Sadness**				
Young	2.29	0.27	0.11	0.91
Older	2.25	0.28		
**Arousal**				
Young	4.32	0.24	0.91	0.37
Old	4.01	0.25		
**Relaxation**				
Young	3.38	0.27	0.31	0.76
Older	3.50	0.28		
**Activity difficulty**				
Young	3.06	0.28	1.55	0.13
Older	2.44	0.29		
**Attention capture**				
Young	4.69	0.22	2.44	0.02
Older	3.92	0.23		

*Data are shown separately for the young and older groups.*

There was no significant difference between the young and elderly people in the judgment of the rate of passage of time (*p* > 0.05). Additional analyses also obtained no significant differences on this measure with respect to the day [*t*(34.69) = 0.90, *p* = 0.38] and the alert time [*t*(33.08) = -0.17, *p* = 0.87]. Responses were also similar in the two groups for emotion-related questions and the difficulty of activity performed at the time of the alert (*p* > 0.05). The extent to which the activity at the time of the alert captured the participant’s attention was however, significantly lower for the old than the younger group (*p* = 0.02).

We next analyzed some potential predictors of PoTJs (shown in **Table 2**). First, we used two composite measures of DJs. These were the mean of verbal estimates for the three stimulus durations, and the mean time produced for the three target durations. The mean time produced for the 500-ms duration was also included, as there was a significance difference between the young and the older group. In neither participant group was either measure of timing predictive of PoTJs. In other words, the verbal estimation and the production of durations did not vary with the experience of time passage at the moment of the alert (all *p* > 0.05).

**Table 2:** Potential predictors of passage of time judgments.

Predictor	Estimate	Standard Error	*t*-value	*p*-value
**Verbal estimation**				
Young	0.00004 [–0.0005, 0.001]	0.0002	0.20	0.85
Older	-0.0001 [–0.0006, 0.0004]	0.0002	-0.46	0.68
**Production**				
Young	0.0002 [–0.0005, 0.0009]	0.0004	0.61	0.55
Older	-0.00001 [–0.0004, 0.0004]	0.0002	-0.05	0.96
**500-ms production**				
Young	0.0002 [–0.0007, 0.001]	0.0005	0.52	0.60
Older	-0.0002 [–0.0007, 0.0004]	0.0003	-0.68	0.50
**Happiness**				
Young	0.28 [0.17, 0.39]	0.06	5.06	0.0001
Older	0.12 [–0.007, 0.23]	0.06	1.94	0.05
**Sadness**				
Young	-0.23 [–0.35, -0.11]	0.06	-4.16	0.001
Older	-0.13 [–0.22, -0.04]	0.05	-2.93	0.004
**Arousal**				
Young	0.21 [0.10, 0.32]	0.05	3.97	0.0001
Older	0.02 [–0.08, 0.13]	0.05	0.44	0.66
**Relaxation**				
Young	-0.19 [–0.27, -0.11]	0.04	-4.92	0.0001
Older	-0.003 [–0.09, 0.085]	0.04	-0.08	0.94
**Activity difficulty**				
Young	-0.04 [–0.16, 0.08]	0.06	-0.67	0.52
Older	0.07 [–0.007, 0.15]	0.04	1.96	0.07
**Attention capture**				
Young	0.10 [0.02, 0.18]	0.04	2.49	0.02
Older	0.08 [0.006, 0.16]	0.04	2.28	0.03

*The predictor is shown along with its associated coefficient value [confidence interval], *t*- and *p*-value. The timing measure predictors are shown with higher precision than the others as with 2 decimal places they would all be rounded to zero. Data are shown separately for the young and older group.*

In contrast to the dissociation between PoTJs and measures of DJs, many variables measured at the time of the alert were predictive of PoTJs. Positive affect was significantly positively related to PoTJs for both the young and older groups (*p* < 0.05). Indeed, for both young and elderly people, the passage of time was judged to go faster when happiness increased. Similarly, negative affect was significantly negatively related to PoTJs in both groups such that the time was judged to pass slower when the state of sadness increased. Reported arousal was also positively related to PoTJs and reported relaxation negatively related, but only for the young group. There was no significant effect of this arousal variable in the older participants. In addition, the extent to which the activity captured attention at the moment of the alert was positively and significantly related to PoTJs for both the young and the older groups, while no significance was found for the judgment of the difficulty of the activity for both groups.

We next used the variables listed in **Table 2** from happiness to attention capture to try predict verbal estimates (**Table 3**) and the times produced (**Table 4**) for the young and older participants taken separately. This made a total of 24 analyses (six predictors × two timing measures × two groups). Of these 24, only one was significant at 0.05, and this was the “attention capture” for the younger group, such that the more their attention was focused on the activity that they performed at the time of the alert, the shorter their temporal estimates. As indicated **Table 3**, there is obviously a significant relationship between verbal estimates and time produced: the longer estimates in the verbal estimation task, the shorter the time produced in the temporal production task.

**Table 3:** Potential predictors of performance on the verbal estimation task.

Predictor	Estimate	Standard Error	*t*-value	*p*-value
**Production**				
Young	-0.64 [–0.81, -0.47]	0.08	-7.73	0.0001
Older	-0.63 [–0.84, -0.43]	0.10	-6.18	0.0001
**Happiness**				
Young	-1.99 [–0.27, 0.23]	13.17	-0.15	0.88
Older	-54.11 [–116, 8.24]	31.56	-1.71	0.09
**Sadness**				
Young	-7.05 [–33.05, 18]	13.24	-0.53	0.59
Older	-13.42 [–21, 33]	30.69	-0.44	0.67
**Arousal**				
Young	6.04 [–21, 33]	12.46	0.49	0.64
Older	-21.70 [–64, 21]	21.84	-0.99	0.32
**Relaxation**				
Young	0.11 [–18, 18]	9.46	0.01	0.99
Older	-26.81 [–66, 12]	20.01	-1.34	0.18
**Activity difficulty**				
Young	1.37 [–13, 16]	7.50	0.18	0.85
Older	26.88 [–17, 70]	21.41	1.26	0.22
**Attention capture**				
Young	-16.20 [–32, -0.13]	7.68	-2.11	0.05
Older	21.37 [–16, 59]	18.91	1.13	0.26

*For each group, the predictor is shown along with its associated coefficient value [confidence interval], *t*- and *p*-value.*

**Table 4:** Potential predictors of performance on the interval production task.

Predictor	Estimate	Standard Error	*t-*value	*p*-value
**Happiness**				
Young	-0.54 [–17, 15]	7.67	-0.07	0.94
Older	34.47 [–4, 73]	18.29	1.88	0.08
**Sadness**				
Young	-2.72 [–25, 19]	10.02	-0.27	0.79
Older	0.63 [–37, 38]	17.48	0.04	0.97
**Arousal**				
Young	6.24 [–8, 21]	6.79	0.92	0.38
Older	16.76 [–5, 39]	10.33	1.62	0.13
**Relaxation**				
Young	-2.75 [–18, 13]	6.55	-0.42	0.69
Older	-3.83 [–29, 22]	11.99	-0.32	0.76
**Activity difficulty**				
Young	2.48 [–5, 10]	4.17	0.60	0.55
Older	4.04 [–9, 18]	7.12	0.57	0.57
**Attention capture**				
Young	9.22 [–2, 20]	5.30	1.73	0.11
Older	-11.42 [–28, 5]	8.10	-1.41	0.18

*For each group, the predictor is shown along with its associated coefficient value [confidence interval], *t*- and *p*-value.*

## Discussion

The results of our study replicated those found by Droit-Volet and Wearden (2015) by demonstrating that PoTJs did not significantly differ between young and elderly people, a result contrary to the conventional idea that time passes more quickly as we get older. Our results confirmed that the experience of time passage in the everyday life did not fluctuate with age but, rather, with the individual’s emotion states. When the participants felt happy, they reported higher rate of time passage. Conversely, when they felt sad, time seemed to drag. In addition, our results suggested that the young and older individuals experienced an acceleration of time passage with the increase in the focus of attention on the current activity. Droit-Volet and Wearden (2015) only found this result for young people, possibly related to the great variability in activities performed by elderly people compared to young people who were all students. However, we found a positive relation between PoTJ and attention capture by the activity performed at the moment of the alert, whereas Droit-Volet and Wearden (2015) found a negative relation. The translation of effect of attention capture by the activity in terms of passage of time thus seems more inconsistent in individuals than emotional effects. Nevertheless, whatever orientation in the speed of time passage (speeding up or slowing down), our study, like that conducted by Droit-Volet and Wearden (2015) and by Droit-Volet (in press a) in participants older than 75 years demonstrated that emotion and attention are the two main factors at the origin of fluctuations of subjective experience of time passage.

The original contribution of the present study is that it also tested judgment of durations at the same time as the participants reported their experience of time passage. The results found no significant relation, or any relation approaching significance, between these two forms of time judgment. Neither the verbal estimates nor the times produced in the temporal production task covaried significantly with the PoTJs. It seems therefore that the subjective feeling that time passes more quickly or slowly than the normal rate of external clocks does not depend on the number of pulses accumulated by a potential internal clock that might provide the raw material for the representation of durations. The duration perceived can change with the acceleration of the internal clock mechanism (for a review see Droit-Volet et al., 2013), without people feeling that time passes more quickly. Conversely, participants may feel that the time passes more quickly without modification in the rhythm of internal mechanism which forms the basis of the perception of duration. This is entirely consistent with the studies on patients with depression which have shown that they express a slowing down of time passage, although they do not exhibit any deficit in the time perception compared to healthy people (Thönes and Oberfeld, 2015), as well as data from the few earlier studies which collected PoTJs and restrospective DJs from the same experiment (e.g., Wearden, 2005, 2008).

Our study using ESM therefore shows dissociation between the judgments of durations and the judgments of passage of time. This dissociation clearly appears in our findings showing that the factors that predicted the individual differences in PoTJ were not significant predictors of judgments of durations both for the verbal estimation and the reproduction task. Indeed, in contrast to the PoTJs, judgments of durations did not vary with the state of happiness or sadness felt at the moment of the alert. Only the factor related to attention paid to the current activity affected these two different forms of time judgment. However, the relation was in the opposite direction for the two types of judgment: the speed of passage of time increased with increasing attention capture while the verbal estimated decreased. Consequently, our results on the prospective time judgments confirmed those found with retrospective time judgments that suggested no evidence that PoTJs and DJs are related (Wearden, 2005, 2008; Wearden et al., 2014).

Other studies need to be conducted before we can definitely confirm the absence of a link between PoTJs and judgments of durations. However, the lack of relation between these two forms of judgment supports the idea that in human beings there are several different types of time judgments. The first one is a judgment of durations common to human beings and to other animals that involves a basic cerebral system functioning as an internal clock. A second is a judgment of passage of time specific to human beings. What determines the subjective experience of a speeding up or a slowing down of the flow of time is still not known for certain, with only a small amount of research to date (e.g., see Flaherty, 1993; Larson, 2004; Larson and von Eye, 2006; Lamotte et al., 2014; Droit-Volet and Wearden, 2015; Wearden, 2015; Droit-Volet, in press a). However, phenomenologists (e.g., Husserl, 1964; Minkowski, 1968/1988) have considered for many years that the judgment of time passage results from individuals’ introspection on their internal life, a “time of self” compared to a “time of world” (Minkowski, 1968/1988) (for a review see Droit-Volet, in press b). The challenge is to scientifically examine this form of time judgment and its impact, if any, on other types of time judgment. Our study provides an initial response to this difficult question: concluding that there is no direct link.

## Author Contributions

Conceived and designed the experiments: SD-V and JW. Performed the experiments: SD-V Analyzed the data: SD-V and JW. Contributed reagents/materials/analysis tools: SDV. Wrote the paper: SD-V and JW.

## Conflict of Interest Statement

The authors declare that the research was conducted in the absence of any commercial or financial relationships that could be construed as a potential conflict of interest.
